# Building climate resilience: awareness of climate change adaptation in German outpatient medical practices

**DOI:** 10.1186/s12913-024-10664-2

**Published:** 2024-02-09

**Authors:** Nicola Alexandra Litke, Regina Poß-Doering, Valeska Fehrer, Martina Köppen, Stephanie Kümmel, Joachim Szecsenyi, Michel Wensing

**Affiliations:** 1 Department of General Practice and Health Services Research, University Hospital Heidelberg, Im Neuenheimer Feld 130.3, 69120 Heidelberg, Germany; 2 Poliklinik Für Zahnerhaltungskunde, Department for Translational Health Economics, University Hospital Heidelberg, Im Neuenheimer Feld 130.3, 69120 Heidelberg, Germany; 3 aQua Institute for Applied Quality Improvement and Research in Healthcare, Maschmühlenweg 8–10, 37073 Göttingen, Germany

**Keywords:** Climate change, Climate resilience, Awareness, Primary care, Climate change adaptation

## Abstract

**Background:**

Climate change is seen as the biggest health threat of the twenty-first century. Making outpatient medical practices resilient is therefore crucial to protect vulnerable groups and maintain quality of care. Awareness is a precondition for action. This study aims to explore awareness (knowledge, experience and attention) of climate change adaptation among stakeholders of outpatient medical practices.

**Methods:**

Semi-structured interviews and focus groups with stakeholders of outpatient medical practices were conducted. The qualitative data were analysed in a two-step Thematic Analysis process.

**Results:**

In total, *n* = 40 stakeholders participated in two focus groups and 26 interviews. The findings show a mixed degree of awareness in outpatient medical practices. The spectrum ranged from a passive role with curative acting only, handing over responsibility to others and a low perceived self-efficacy to a proactive and responsible implementation of adaptation strategies. Participants who saw the need and responsibility of climate change adaptation in medical practices perceived low additional workload. In general, implementation of climate change adaptation measures and general awareness of climate change adaptation appeared to be depending on a certain tension for change and a higher self-efficacy.

**Conclusion:**

Medical practices, and specifically primary care, plays a crucial role in climate change adaptation, and awareness needs to be increased further in order to cope with consequences of climate change. To facilitate this, there should be a strong emphasis on climate change adaptation strategies being part of outpatient care provider roles rather than being perceived as an “add-on” to already high workloads.

**Supplementary Information:**

The online version contains supplementary material available at 10.1186/s12913-024-10664-2.

## Background

Climate Change (CC) is seen as the biggest health threat of the twenty-first century [1] implying extensive impacts on population health and consequences throughout health systems [2]. Outpatient medical practices and in particular primary care practices, are affected by these consequences as they are often the first and main point of contact for vulnerable groups such as patients with chronic diseases [3]. Progress in CC continues dramatically and consequences have worsened with other co-existing crises such as energy-crisis and COVID-19 [4]. Making outpatient medical practices resilient is therefore crucial to protect vulnerable groups and maintain quality of care throughout a changing environment.

The World Health Organization defines climate resilient health care facilities as “[…] institutions that anticipate, respond to, cope with, recover from and adapt to climate-related shocks and stresses, while minimizing negative impacts on the environment and leveraging opportunities to restore and improve it, so as to bring ongoing and sustained health care to their target population and protect the health and well-being of future generations” [5]. While this blueprint is designed primarily for hospital settings [6], approaches that focus on the outpatient sector are rare. However, elaboration of potential adaptation strategies gains weight.

In the context of CC, several definitions describe the process of adaptation. Adaptation measures are defined as management of the unavoidable CC consequences and as strategies that are “[…] implemented in response to the immediate consequences of climate change and to help reduce the adverse effects of these on human health and wellbeing” [7]. The United Nations pathway to build climate resilience names Awareness-raising and advocacy as step one in the adaptation process [8]. Consistent with this, ( awareness is listed as the first of five adaptation stages [6]. Iturriza et al. 2018 [9] enhance the United Nations International Strategy for Disaster Reduction (UNISDR) definition of awareness in the context of CC and understand it as “the result of the interaction between three mechanisms: experience, attention and knowledge” [9].

As CC is described as a global health crisis, the project RESILARE strives to assess and foster climate resilience in German primary and ambulatory healthcare. To support preparation of outpatient medical practices for crisis situations and to increase their crisis resilience, related quality indicators were developed and piloted with primary care providers [10]. Within RESILARE, this present study aimed to assess awareness (knowledge, experience and attention) of outpatient medical practices about the consequences of CC on population health and explore adaptation strategies pursued to counteract.

## Methods

This study is part of the first phase of the project RESILARE. Within this first study phase, a qualitative study was conducted including semi-structured interviews and focus groups with stakeholders in outpatient medical practices [10].

The study presented in this manuscript was approved by the ethics committee of the Medical Faculty of the University of Heidelberg, Germany, (S-456/2021). All participants gave written informed consent prior to data collection.

### Study sample

A purposive sample of 40 stakeholders in German outpatient medical practices was planned. Invited for participation were outpatient care physicians, medical assistants, quality managers, practice managers, hygienic managers, scientists experienced in outpatient medical practices, quality indicator and/or CC experts, as well as stakeholders on health system level experienced in outpatient medical practices. Only participants older than 18 years and able to give written consent were included. The sampling strategy aimed at variety of geographical locations through Germany, practice sizes, different forms of practice organization (networks, single practices, and other), medical disciplines and within the specific profession of the experts.

Recruitment was conducted across the networks of the study partners and study advisory board as well as the newsletter of the aQua Institute for Applied Quality Improvement and Research in Health Care GmbH. Further study partners are a German medical care centre, a German care network and an institute for quality certification in medical practices. Invitations were sent via mail, e-mail, phone, or participants were invited personally. Additionally, snowball sampling was applied. If participants were affiliated to the same network or institution such as a primary care practice, they were asked to participate in a focus group. All other experts participated in single telephone interviews.

### Data collection and measures

For qualitative data collection, focus groups and interviews were conducted from 12th July until 14th October 2021. Focus groups were done via an online video conference, where all participants used cameras and microphones. Interviews were conducted using a telephone.

Interviews and focus groups were based on a semi-structured interview guide and included open questions.

The interview guide was developed based on a literature research on crisis resilience in healthcare and climate resilience and the resulting definition of climate resilience by the WHO [11]. The interview guide was developed by a group of researchers and experts (authors as well as further researchers from University Hospital Heidelberg, Germany) with expertise in health services research, qualitative research, work or quality management experience in medical practices, CC and health, as well as a researcher who focused on the coping of German general practices with the Covid-19 pandemic. Table 1 gives an overview of themes and subthemes addressed within the first phase of the RESILARE-project [10, 12].

**Table 1:** Topics addressed in semi-structured interviews and focus groups

**Subject**	**Subthemes**
General, organisational resilience	• Awareness of current and future crises • Reaction, responses and coping strategies as well as reflection of these strategies • Recovery from past crises, lessons-learned • Preparedness and identification of potential future crises • Determinants for promoting crisis resilience in primary care practices
CC adaptation	• Knowledge and experience of CC consequences on health • Respondence and coping strategies for CC adaptation
CC mitigation	• Attitudes/roles and beliefs of CC mitigation • Known and experienced mitigation strategies • Determinants for future implementation of mitigation strategies
Quality indicators	• Ideas and wishes for future quality indicators for crisis-resilience • Determinants for implementation of quality indicators

The same questions were asked for all different professions. However, the exact wording of the questions slightly varied between participants currently working in medical practices and other stakeholders. All participants were also asked to fill in a form addressing their sociodemographic characteristics sent to them by mail after obtaining consent for participation. This also included consent for audio and video recording.

Focus groups were conducted using the secure video-meeting platform heiCONF of the University of Heidelberg. The web conferences were video and audio recorded, the interviews were audio recorded. Handwritten notes were taken during interviews and focus groups. All audio and video records were transcribed verbatim. Focus group transcripts also included non-verbal reactions of the study participants such as nodding or signing thumbs-up. No interview or focus group was repeated or cancelled and no transcript was returned to the participants for correction. Interviewers recommended to be in a quiet and private place where participants felt comfortable during interview or focus groups.

Data collection was done by NL and VF. NL is a female researcher and doctoral candidate with experience in qualitative research and a background in health services research. VF is a female then masters-candidate and trainee in the RESILARE-project.

### Data analysis

Qualitative data were analysed in a two-step process according to the Thematic Analysis of Braun and Clarke [13]. First, all transcripts were coded inductively by NL and RPD. RPD is a female researcher with a background in health services research and is an experienced expert in qualitative research.

After the initial coding, a focused literature research was done in April 2023 on climate resilience, adaptation and awareness. The theory of Iturriza et al., 2018 [9] on CC awareness was then used to categorise initial codes. In a second step, all codes were recoded accordingly. Furthermore, word searches for “climate*”, “heat*” and “environment*” were performed, as well as “ventilat*” and “air condition*” to complement data analysis of previously developed codes in the category of attention.

During the entire process of data analysis, the researchers held meetings on a regular basis and discussed the coding process regarding specific codes, transcript passages, and resilience definitions.

All transcripts were analysed as a whole, although not all interview questions were related to the research questions of this study. Arguments explicitly referring to CC adaptation were considered in the second step of analysis. General attitudes or non-explicit arguments with reference to the research questions are reported as such.

Data management and analysis was conducted using MAXQDA software Version 20 (Verbi GmbH). IBM® SPSS Statistics Version 26 was used for analysis of sociodemographic data.

## Results

In total, *n* = 40 stakeholders participated in two focus groups and 26 interviews. During data collection, participants were at their workplace, commuting or at home. The socio-demographic data collection shows that most participants were female (*n* = 24), working in a medical practice as physician (*n* = 14) or medical assistant (*n* = 16), and 25 to 39 years (*n* = 14) or 40 to 59 years old (*n* = 17). No participant dropped out. Further professions included researchers, health system level employees, social workers, pharmacists and other health professionals. Medical assistants had additional specialised training as care assistants in general practice, non-physician practice assistants, quality managers or hygiene managers.

Participants working in practices reported first-hand experiences and second-hand experiences of colleagues or friends. Participants from other parts of the health system (n = 10) worked in research, health insurance, or politics and reported their experiences from direct interface with outpatient medical practices and practice staff in Germany during crises such as the swine flu, COVID-19 pandemic or extreme weather events.

**Fig. 1:**
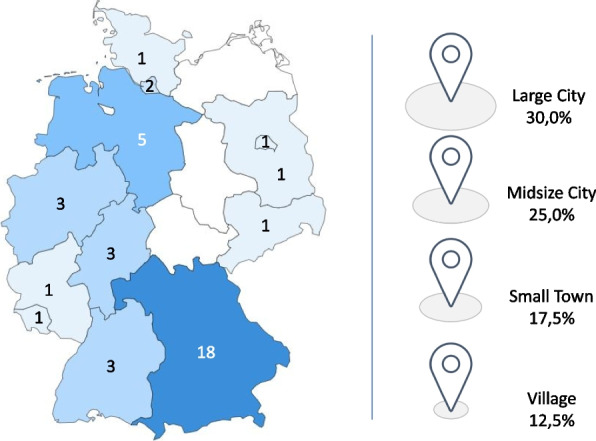
Geographical location of participants

Geographically, practices and participants were located throughout Germany (south, east, west, north) in rural, urban and sub-urban areas (see Fig. 1). Practice size varied between single practices with a team of two and practice networks with 40 employees. Participating practices specialized in general practice, internal medicine, neurosurgery, pneumology, dermatology, orthopaedics, otorhinolaryngology and gynaecology.

### Impacts of climate change on health and healthcare

#### Knowledge of climate change impacts on health

Participants primarily named extreme weather events such as heatwaves and torrential rain with flooding as possible CC consequences that might affect health. Medication was considered to have lower or different effects in higher temperatures, patients were assumed to stay at home more during a heatwave or a blizzard. Respiratory diseases such as asthma or COPD, other chronic diseases such as arthrosis, backpain or gout as well as allergies were perceived to be on the rise because of changing weather conditions and longer, more intense seasons. Infectious and vector-borne diseases were named as consequences as well. Some participants associated the COVID-19 pandemic with CC and expressed their concern that more variants could occur in the future. Migration was expected to increase, implying new tropical diseases as well. Besides expected resource shortages regarding staff, material and medication, energy and nutrition, the need for healthcare was expected to increase and result in an increasing lack of resources. Deteriorating mental health was also discussed. While there was a variety of mentioned possible consequences, this list was composed of statements made by only a few and most participants referred to heatwaves only.

Interviewer: “Which further consequences of climate change do you see for medical practices?”

Participant: < long pause > “Nothing comes to mind now“ (Int17/female/medical assistant)

#### Experience of climate change impacts on health

In general, with CC, working conditions were perceived as increasingly difficult. Prevailing heat in practice premises during summer was perceived as reducing efficiency and implying stress. Some described that during a heatwave, they were not able to continue to work, had to take a break or “go on *strike*” (Int15/female/medical assistant) and go home, or “… *had no consideration for the patients”* (Int17/female/medical assistant) since they were preoccupied with themselves then*.* Some participants reported that they and their colleagues felt a decrease in enjoyment of work and thought about leaving their job in primary care practices more often. This was seen as contributing to the lack of workforce which was perceived as a factor for worsening working conditions. With high temperatures in the practice, medical devices were described to fail and diagnostics or treatments needed to be cancelled.

*„Yes, the ultrasound device overheats eventually as well.“* (Int13/female/medical assistant)

When practice procedures continued as usual, poor patient conditions related to heat stroke, sun burn, cardiovascular problems, and vertigo were experienced. Patients were perceived to be “[…] *in poorer condition during operations”* and sometimes emergency had to be called *“[…] because of blood pressure”* (Int03/female/medical assistant).

Some participants perceived that patients were more likely to cancel appointments and stay home during a heatwave or other extreme weather events like a blizzard, resulting in insufficient use of medication because prescriptions were not picked up and filled at a pharmacy. This was also described to result in economic losses for the practice during so called “*summer slumps”* (Int13/female/medical assistant). Few participants described little or no experiences with heatwaves. Further perceived extreme weather events included floods which were discussed as second-hand experience.

„I mean, we experience it already now. So, we have extremely hot summers with all kinds of health and economic problems. During these hot summers, turnover is simply breaking off. People don’t leave the house, you just notice that.“ (Int05/male/pharmacist)

A few participants from practices specialized in tropical or travel medicine described experience with a small number of cases with vector-borne diseases. Participants often considered such diseases to be a possible CC consequence, yet felt these did not play a role in their work in healthcare yet and referred only to patients catching them while vacationing in other countries. With longer seasons and warmer winters, increases in patients with tick bites and allergies were described. With changing weather conditions, participants felt that patients reported more problems with backpain and respiratory diseases such as COPD and Asthma.

„Yes, because there are significantly more people suffering from allergies than before, no doubt, they also start earlier and later in life. Even at a higher age, where you would think they don't develop an allergy they actually still develop one and seasonally, they just start much earlier and of course suffer longer.” (Int14/female/medical assistant)

#### Attention to effects of climate change on health and health care

With regards to CC consequences, some participants stated rather generally that “*no one really knows what the consequences are, it depends on where you are*” (FG2, Interviewee 4/male/physician), or “*this* (climate change) *will have an impact on health, for sure*” (Int11/female/medical assistant). A small number of participants talked about experienced CC consequences such as heat or flood but did not mention CC explicitly. Others stated they had no time at all to even think about CC because there were enough other problems they had to face. One person rated CC adaptation (and mitigation) as “the most irrelevant topic in our work” (Int10/female/medical assistant). Some experienced heatwaves as crises, attributing this to no air condition or fans being available in their practice or being too short on staff to respond to patient volume and needs. Most participants stated that heatwaves posed no problem for them, because air conditioning was available on-site and therefore patients felt comfortable during their stay. Ambivalent and confounded statements were observed when no or only little attention to experiences with CC or heatwaves were discussed, yet drastic influences on care provision like dysfunctional medical devices were described to occur during phases of intense heat and closing the practice as re-active attitude. Referring solely to heat and a few corresponding basic adaptation strategies, one person rated personal awareness as high. Another felt heat and drought were common in their region and the topic was irrelevant and no problem at all because people in the region were used to heat.

Most participants shared that they perceived a low CC awareness within their own practice and in other practices. Some observed a slight increase over recent years and only a few saw a high awareness in their own practice. Most statements related to having to deal with heat in practice premises because of no air conditioning. A few participants felt that CC progressed slowly and consequences were not experienced intensely enough yet. Some described fear and anxiety related to CC observed in staff and patients and promoted a gentle approach to the topic towards patients.

“I simply just try, so many fears are stirred up, also whether it is now over the television, which is very present with the patients we have in the family doctor's practice, then I also simply try with good > laughs< arguments to pass this on and not to stir up more fears”. (Int01/female/medical assistant)

Regarding awareness referring to CC consequences, a relation between first- or second-hand experience was observed in the data. Participants described that personal affinity to the topic increased awareness. If someone was directly affected by a CC consequence, this person considered it relevant for primary care, felt engaged with the topic, and named adaptation strategies. Some expressed that CC consequences only affected others, not themselves or their work yet, but could become a topic in the future that did not worry them since Germany had a good health system. Others attributed a minor role to CC consequences, because at the time, temperatures were not high outside or there were less heatwaves than in years before. Proactive efforts to increase attention levels were not contemplated.

“…only during summer, when it is hot for one or two weeks, okay, but right now, this plays a minor role” (Int19/female/medical assistant)

### Adaptation strategies

Findings are related to experienced adaptation strategies, though some strategies were not explicitly named as first-hand experience. Table S1 (supplementary material) summarizes all adaptation strategies named and experienced first-hand or second-hand in primary care.

#### Experienced strategies

Primarily, experienced and practiced adaptation strategies referred to heatwaves. Some participants could not name any strategies without asking for an example, and some only named mitigation strategies for reducing CO_2_ footprint of the practice.

Installing air-conditioning or using a fan were the most commonly discussed and practiced solutions, followed by drinking and offering water to patients or staff in a worsening general condition. Some participants mentioned advice they gave during a heatwave to protect vulnerable patients included avoiding direct sunlight, healthy diet and avoiding hard work. Some reported not giving any advice or acting only when a patient with heat-related stress consulted them. Access to sufficient resources such as staff was named as another adaptation strategy. Some participants described, they perceived a heatwave as a crisis, because there were not enough staff to conduct adaptation strategies.

“In any case, the lack of staff, um, that we simply had too few staff, that we could say or that the doctors could say, we are now sending a care assistant out, who will then measure the patient's blood pressure or take blood samples” (Int20/female/medical assistant)

Some participants named strategies they wished would be practiced in the future, but had not experienced them yet. These included government initiated categorizing of heatwaves as natural catastrophe to facilitate access to resources and local networking (e.g. with red cross or fire department). While no participant knew of any official action plan or institution that published one, action plans and structured recommendations on how to act before and during a heatwave were desired by most participants. One participant described knowing a General Practitioner who had developed and implemented an action plan for his practice by himself.

#### Attention to adaptation measures

When talking about CC adaptation, some participants jokingly devalued the topic and mentioned they were “happy about warm weather” (Int09/female/medical assistant) and would “get ice cream during work” (Int10/female/medical assistant). One General Practitioner rated CC as an esoteric and provoking topic closely linked to activism and political discussions where evidence referring to potential consequences was still missing. This participant felt adaptation measures needed to be evidence-based and communicated to physicians neutrally in a scientific style to effectively promote implementation of adaptation measures.

When asked for further ideas beyond practiced or known adaptation measures related to heatwaves, participants stated they didn’t know any or again named mitigation measures. As a contrast, when asked about mitigation measures, a number of participants first answered they had never thought about it, but then continued to generate ideas and reflected on numerous strategies and possibilities to reduce CO_2_ footprint of their practice. Some participants thought to have no impact as a single person or practice compared to large CO_2_ emitting industrial institutions, thus indicating an imbalanced responsibility and a perceived lack of self-efficacy. Particularly medical assistants described this lack of self-efficacy when emphasizing they had to carry out what they were asked to do by physicians and “*work like puppets*”, “*not think on their own*” and just “*earn money*” (Int15/female/medical assistant). One medical assistant described promoting knowledge about CC adaptation as empowerment that would offer her a new role within the practice team.

Only reactive, directly linked solutions were discussed as adaptation strategies and measures: Air conditioning was seen as primary solution to heat, dizzy patients or staff were recommended to drink water. Preventive, proactive actions and thoughts “outside the box” were rare: should a practice be destroyed by flood, participants saw no possibility at all to continue providing health care in any other way. Most participants viewed CC adaptation only from their own perspective leaving patient and staff needs, or the needs of other practices in other regions of Germany unattended. One medical assistant described the need for patient surveys so that physicians in her practice would start to see their needs and would buy some water for staff and patients which they refused to do so far (Int03/female/medical assistant). When a practice was cooled down during a heatwave, there was no problem seen at all and patients were rated to feel comfortable. Consideration of circumstances outside the practice, for instance regarding living conditions of vulnerable patients, was not voiced. Some participants only saw heatwaves as a crisis in case of too many emergencies they could not handle anymore or if emergency kits were defect, thus putting stronger emphasis on the implied higher workload than on CC implications themselves. A reactive and curative behaviour was also observed by participants themselves in colleagues.

“That is the bad thing. It first has to get to the point where a person wakes up, and then it's usually too late.” (Int15/female/medical assistant).

Some participants stated it was not in their responsibility and they had no time at all to implement adaptation measures such as warning vulnerable patients in advance or educating them about possible threats caused by CC. Willingness to assume a role in this regard was questioned since physicians “*concentrate much more on medicine*” (Int09/female/medical assistant) and practices had to cope with extremely high workloads leaving no additional capacities to fall back on in a crisis. A low cost–benefit ratio was described for preventive measures, so only cheap measures would be considered as preventive measures in general were considered to not materialize immediately.

„Well, so I think the doctors' practices also want to stick to their (...) already assigned role usually. I believe that very few physicians are now somehow prepared to take on additional roles.” (Int04/female/researcher and physician)

Others saw themselves and primary care in general as responsible for the protection of vulnerable patients and described they had already sustainably implemented preventive measures like warning patients before a heatwave or conducting home visits for vulnerable patients affected by heat. These measures were described as practical and not perceived as significant additional workload. These participants rated CC as an unavoidable crisis that affects and will affect everyone and showed a high tension for change because they wanted to protect vulnerable patients and future generations from CC consequences or described any progress related to CC as too slow.

“Yes, it's a lot, but we just tell ourselves that if we talk to them now, […], then the one minute doesn't matter anymore. And then we prefer to take our time instead of having a big mess afterwards.“ (Int20/female/medical assistant)

## Discussion

Findings in this study show a heterogeneous awareness of CC consequences on health and adaptation strategies in outpatient medical practices in Germany, even though every participant had experienced effects of CC consequences, mostly by heat. The spectrum of awareness across the highly diverse sample ranged from a passive role with curative acting only, handing over responsibility to others and a low perceived self-efficacy to a proactive and responsible implementation of preventive adaptation strategies. Participants who saw the need and responsibility of CC adaptation in outpatient medical practices, perceived no additional workload implied with such strategies. In general, implementation of CC adaptation measures and general awareness of CC adaptation appeared to be depending on personal affection, a certain tension for change and a profound perception of self-efficacy.

These findings are consistent with the observation of a lower perceived personal affection as well as psychological distance to CC consequences on health within the German public [14]: Consequences played a minor role and only few participants described themselves as part of the vulnerable group even though they fulfilled specific criteria like higher age [14], a psychological phenomenon described as a division of available knowledge, affection and acting [15, 16]. This supports the importance of care providers’ awareness to protect public health and help promote public awareness of CC consequences on health.

In some participants, a missing link between practiced medicine and CC adaptation was assumed. Furthermore, the willingness of taking on another role was perceived as low as workload was already described as high. However, medical staff is considered crucial in successful CC adaptation and protecting public health [17]. Particular care for vulnerable patients as well as preparedness of the health system are part of the WHO heat-health action plan that is expected to prevent adverse health effects of heat-waves and hot weather largely if implemented adequately [18]. Therefore, CC adaptation should be promoted as part of the current role as physician or medical assistant rather than as another “additional role” as it was perceived by some in this study. A first step could be to include CC adaptation in basic and continued training programmes for healthcare workforce and physicians alike [5].

All participants described to be affected by CC consequences in some way, including economic losses for practices during periods of particularly high temperatures, yet awareness was observed to vary throughout the sample. Some participants could not name any adaptation strategy unless an example was provided, and some responded with naming only mitigation strategies for reducing CO_2_ footprint of the practice. This might imply a lack of knowledge and experience in CC consequences and appropriate adaptation strategies and a higher awareness about mitigation measures. However, it is inevitable to be aware of and understand the underlying problem to be able to take action [9]. In general, personal affection is described to raise awareness and hence the willingness to take action [9]. As outpatient medical practices play a crucial role in preventing vulnerable groups from CC health-consequences, knowledge about adaptation strategies needs to be promoted. This is not only suggested by the participants in this study, but also described in literature [19, 20].

Public engagement with health aspects of CC is at an all-time high and media presence increased steadily [4]. Data collection for this study was conducted in 2021, which might indicate a certain outdatedness of findings. However, the United Nations Climate Change Conference (UNFCCC) has its roots in 1979 and the call for action through the WHO heat-health action plan guidance was published in 2008 already. So far, standardized and effective strategies like national heat action-plans are still lacking [21]. A PubMed search using the term “"awareness" AND "climate change adaptation"” in June 2023 resulted in 59 articles and the oldest was published in 2010. Also, as recent as June 2023, the first national heat action-day was conducted in Germany [22] and the Federal Ministry of Health published a provisional national heat action plan addressing the German health sector [23]. Hence, CC adaptation awareness is assumed to have increased within the last few years, which could be observed within this study as well and is consistent with Álvarez-Nieto et al. 2022 [24]. Internationally, progress in CC adaptation is observed, too [4, 24, 25]. However, despite this progress, the pace of CC adaptation is described as “far from what is necessary to reduce the health impacts of climate change” [4] and long-term implementation of heat-health action plans is described to remain challenging [25]. Regarding the construct of awareness, sensitivity to change can be compared: media campaigns, taxation and structured prevention plans have been implemented internationally to raise awareness and reduce the use of tobacco since at least 20 years. Still, in 2020, 22,3% of the global population used tobacco, resulting in eight million deaths per year [26].

Findings of this study show that poor working conditions caused by CC consequences result in staff considering professional redirection as coping with conditions becomes more difficult. In the long term, this might contribute to an increased lack of health workforce in outpatient medical practices which again is perceived as a crisis by these participants [10].

Within the project RESILARE, CC is analysed as part of an overarching disaster research [10, 12], which is seen as “particularly germane because many of the projected impacts of CC will take the form of acute and longer term natural disasters” [27]. To complete the construct of climate resilience, it is necessary to look into CC mitigation as well [5]. Findings in the RESILARE project concerning mitigation were described already [12], yet further studies should broaden the knowledge base regarding mitigation. All identified determinants should be considered for implementation strategies of national mitigation and adaptation plans.

### Strengths and limitations

It is necessary to point out that the study information detailed that heatwaves or CC would be among discussed topics. Therefore, it is possible that participants named heatwaves as an example for crisis situations, along with COVID-19. However, interview themes were summarised and propagated primarily using the term “crisis resilience”, so most of the participants focused on crisis resilience in general. The sample did not only consist of experts and only half of the participants named heatwaves as a current or future crisis spontaneously during the interview.

Great importance was attached to a high variance within the study sample during the recruitment process. Density of the data generated in telephone and video-based interviews was similar and allowed thematic saturation to be achieved. Therefore, and according to empirical guidance, sample size is considered adequate [28]. CC adaptation was one of several discussed topics in the interviews. Although socially desirable answers are possible, findings show no such trend.

As the interviewees were also invited through the networks of all study partners, NL knew some participants in person in a professional context. However, this had no influence on the use of the interview guide and wording of the questions.

The conduction of the study is reported according to the Consolidated Criteria for Reporting Qualitative Studies (COREQ) checklist [29].

## Conclusion

Outpatient medical practices play a crucial role in CC adaptation, and awareness needs to be increased further in order to cope with steadily worsening CC consequences. To facilitate a pro-active culture, there should be a strong emphasis on CC adaptation strategies being part of healthcare provider roles rather than being perceived as an “add-on” to already high workloads. A starting point for raising awareness could be found in continued medical training courses, healthcare curricula and scientific conference contributions. Target-group specific materials and inputs will have to be developed.

### Supplementary Information

**Additional file 1:** **Table S1.** List of discussed and experienced adaptation strategies in Outpatient medical practices in Germany.

**Additional file 2.** COREQ (COnsolidated criteria for REporting Qualitative research) Checklist.

## Data Availability

Selective data and interview guides are available in German language on reasonable request from the corresponding author.

## Abbreviations

CC: Climate Change
WHO: World Health Organization
