# Supplementary material for: Building climate resilience: awareness of climate change adaptation in German outpatient medical practices
Source: BMC Health Serv Res. 2024 Feb 9;24:184. doi: 10.1186/s12913-024-10664-2 (PMC10858569; doi:10.1186/s12913-024-10664-2)
Supplement: Supplementary file 1 — Additional file 1: Table S1. List of discussed and experienced adaptation strategies in Outpatient medical practices in Germany. [file 12913_2024_10664_MOESM1_ESM.docx]

**Supplementary material**

Table S1: List of discussed and experienced adaptation strategies in Primary Care Practices in Germany

| **In general** | |
| --- | --- |
| awareness | - Promoting awareness and knowledge about the consequences of climate change e.g. through trainings or quality management* - Promoting knowledge about adaptation strategies e.g. through trainings or quality management* |
| preparedness | - Conducting a general risk assessment for possible hazards like flood or heat* - Linking the practice to warning systems (ozone, UV index, heat, pollen, extreme weather events in general and other crises)* - Ensuring flexible and sufficient access to resources*   - Sufficient workforce   - An appropriate amount of regularly used medication and material in case of a sudden shortage   - Durable and heat resistant medical devices |
| patient centeredness | - Educating patients about consequences of climate change on health (e.g. through climate themed consultations, handouts or brochures) - Conducting a patient survey to identify wishes and needs (e.g. water access in waiting area)* - Ensuring access to the practice in crises situations (like blizzard, flood), and promoting access to treatment or medication for patients affected by the crisis e.g. through teleconsultation/phone, suggesting the use of pharmacy delivery services.* |
| occupational safety and health | - Creating agreeable working conditions - Protecting staff in crises situations like endemics or heatwaves |
| **Heatwaves** | |
| practice level | - Promoting a pleasant indoor climate through   - Air-condition   - fan   - natural ventilation   - closing shutters in advance   - air filter   - offering wet and cold towels / footbath for staff - Offering free access to water for staff and patients - Adapting termination of appointments   - open early, stay open late   - Implementing a longer lunchbreak when peak daily temperature is reached   - Treat vulnerable patient groups in early mornings/late evening   - Reschedule appointments that require a cooler room climate (e.g. ultra-sonic scans, operations, vertigo diagnostics) - Reducing working hours of staff, planning more work breaks - Promoting an adequate/cool storing of medication and medical devices |
| patient level | - Informing and warning patients about upcoming heatwaves   - Call vulnerable patients   - Contact nursing homes   - Hand out information sheets in the practice* - Giving advice about how to react to a heatwave   - Drink water   - Stay inside / in cooler places   - Only go outside in the early mornings/late evenings   - Avoid food that is hard to digest   - Avoid hard work   - Avoid working in the sun (e.g. gardening)   - Ask others for help (medication delivery, handling of exhausting tasks)   - Use sun protection - Treat patients at home if possible, e.g. through telemedicine / phone consultation, home visits - Conducting medication checks on a regular basis (e.g. before & after summer) |
| **Flood** | |
|  | - Conducting a risk assessment* - Protecting equipment, e.g. by storing sandbags* |
| **Tropical diseases / vector-borne diseases / infectious diseases** | |
|  | - Protecting staff - Keeping a list of important phone numbers and contacts at hand (e.g. tropical institute, poison control centre) - Promoting prevention through increased vaccination rates* |

* discussed without specific indication of first- or second-hand experience
